# Sex Differences in the Individual Behaviour of Bait-Attracted White Sharks (*Carcharodon carcharias*, Linnaeus, 1758) Are Linked to Different Environmental Factors in South Africa

**DOI:** 10.3390/biology11121735

**Published:** 2022-11-29

**Authors:** Olga Mouteira Azevedo, Ana Mafalda Correia, Primo Micarelli, Francesca Romana Reinero, Giuseppe Rijllo, Gianni Giglio, Emilio Sperone

**Affiliations:** 1 Department of Biology, Ecology and Earth Sciences (DiBEST), University of Calabria, 87036 Rende, Italy; 2 Coastal Biodiversity Laboratory, Interdisciplinary Centre of Marine and Environmental Research (CIIMAR), 4450-208 Matosinhos, Portugal; 3 Department of Biology, Faculty of Sciences, University of Porto (FCUP), 4169-007 Porto, Portugal; 4 The Sharks Studies Centre—Scientific Institute, 58024 Massa Marittima, Italy

**Keywords:** white shark, ethograms complexity, sexual differentiation, environmental influences

## Abstract

**Simple Summary:**

Feared by some and loved by others, the white shark is globally distributed and easily found in some well-known hotspots. The development of telemetry tools has contributed to the increasing knowledge of the movement ecology of these predators. Meanwhile, the cage-diving industry has exponentially grown in the last few years, becoming a platform of opportunity for direct contact with them. Less well understood are the processes that influence the complex activity of a white shark. Using a non-invasive approach, it was demonstrated that different abiotic factors could influence the behaviour of this species. This study brings new insights into how females and males use environmental information to manage their activity and behaviour complexity. In the context of a changing climate, it is important to understand how sharks respond to a fluctuating environment to effectively manage and mitigate human–shark interactions while supporting conservation efforts.

**Abstract:**

The white shark (*Carcharodon carcharias*) is a charismatic species and, consequently, one of the most studied and protected sharks. This species can be found in a wide range of temperatures and depths, showing site fidelity and migrating across the oceans. This offers a challenge to understanding the processes influencing their lifecycle and, more importantly, assessing anthropogenic disturbances to their populations. These predators’ behaviour has been linked to diverse abiotic factors. Here, an ethological approach was used to understand the influence of environmental variables on white shark behaviour. A different environmental impact was found between the activity of females and males toward the bait. Females performed a higher number of behaviours under daylight, lower sea surface temperatures, short wavelets, clear and cloudy skies, under La Niña events, elevated moonlight and high tides. Males behaved with more complexity at dawn, medium sea surface temperatures, large wavelets, few clouds, high tides, and elevated moonlight. The world’s aquatic habitats are experiencing significant physiochemical shifts due to human-induced climate change. Knowledge about how white sharks respond to environmental factors is essential to guide management and conservation actions.

## 1. Introduction

The white shark (*Carcharodon carcharias*) is the world’s largest carnivorous fish, and it is broadly distributed in temperate and tropical waters [1]. This apex predator has received considerable attention, mostly due to its charismatic profile, important ecological role, and vulnerable conservation status (e.g., [2,3,4,5]). These animals routinely migrate thousands of kilometres [6,7], but they can also be easily found in known aggregation areas, such as South Africa [8], Australia [9], and Mexico [10].

When marine species undertake long-distance locomotion or migration in the sea, they become more challenging to study since their lifecycles can only be partially directly observed. Advances in biologging tags have made it possible to track the hidden behaviour of these animals and their environment [11]. Data on spatial and temporal patterns of space use can provide information on aspects of behaviour, sociability, energetics, and predator–prey relations [12]. However, many of the tags require rigid attachment to the animals, and their application on large predatory sharks may have significant consequences for individual survival and behaviour [13]. Different methods have been used to study white shark behaviour, such as direct observation [14], diverse telemetry systems [15], and catches [16]. In the past, direct observation was commonly used either from land or boats [17,18]. The growth of the wildlife tourism industry using bait attraction and cage-diving made closer contact with these animals possible, allowing for an improvement in visual monitoring methods. The use of these non-invasive procedures, coupled with statistical modelling, could be a useful approach for describing the ecology of threatened species such as the white shark [19]. The individual surface behaviour of white sharks is not a simple stimulus–response reflex but rather a complex tactical situation in which animals show plastic responses [20]. Despite some impacts on their activity patterns [21,22], these tourism activities have no significant conditioning on *C. carcharias* behaviour [23], which means they represent a platform of opportunity suitable for research and promotion of the conservation of this species. The predation or approach strategy of white sharks is not well known due to the logistic difficulties and relatively rare chances to observe natural predatory events [24,25]. Therefore, using bait to attract white sharks allows the study of their approaching behaviour to potential prey that has been previously detected.

Sexual segregation has been noticed in white sharks’ occurrence and distribution [15,17], not only in adults but also in juveniles and sub-adults [26]. In the Northeast Pacific Ocean, males make annual migrations, while large, likely pregnant, females migrate biannually [27]. In False Bay, South Africa, females are present year-round, while males are only observed during autumn and winter [26]. In South Australia, the opposite is verified, with males present year-round and females present only from autumn to mid-winter [28]. Both in Gansbaai (South Africa) and Port Stephens (Australia), females and males use their habitat differently, with females spending more time in inshore areas [25,29]. Different drivers related to different physiological needs can lead to segregation in *C. carcharias*. Environmental variables such as sea surface temperature may be related to sexual segregation in this species due to reproductive habits [28].

White shark behaviour has been linked to diverse environmental factors, such as sea surface temperature, turbidity, lunar phase, cloud cover, sea surface salinity, tides, swell, currents, wind, upwelling, and barometric pressure [30]. However, a comprehensive analysis of these interactions from the ethological point of view is missing. In general, the behaviour is considered the animals’ first line of defence in response to environmental changes, and it can influence research findings in unexpected ways [31]. To date, most of the studies involving white shark behaviour and environmental factors have been limited to habitat use (e.g., [32,33]), occurrence (e.g., [19,34]), and predatory activity (e.g., [35,36]). A better understanding of how oceanographic variables influence *C. carcharias* behaviour may help to predict their distribution, response to increasing anthropogenic stressors, and role in ecosystems throughout their geographic range. The knowledge of this predator’s feeding behaviour may help to understand their potential impact on prey [12] and, consequently, on the ecosystem. The ability of the shark to detect and approach the prey and the ability of the prey to avoid attack and subjugation are likely affected by environmental factors such as water clarity, ocean depth, temperature, tidal height, ambient light levels, and currents [30,35]. Activity peaks of predators should be correlated with periods when environmental and biological factors are optimal for exploiting a selected prey item [37]. Environment-related changes in energy expenditure may impact how predators operate and forage [38]. An ecosystem-based approach is important for the protection of the sharks and their prey for the conservation of the top–bottom control roles in their ecosystem. Bycatch and shark–human interaction management also need improvements in order to achieve better conservation outcomes in the face of global climate change. In the present work, we intend to understand the influence of different environmental factors on the individual behaviour of female and male white sharks by applying a non-invasive method and using ethological data. For that, the complexity of the ethograms performed, i.e., the number of behavioural units performed towards a bait under specific environmental circumstances, were analysed and compared.

## 2. Materials and Methods

### 2.1. Study Area

**Figure 1:**
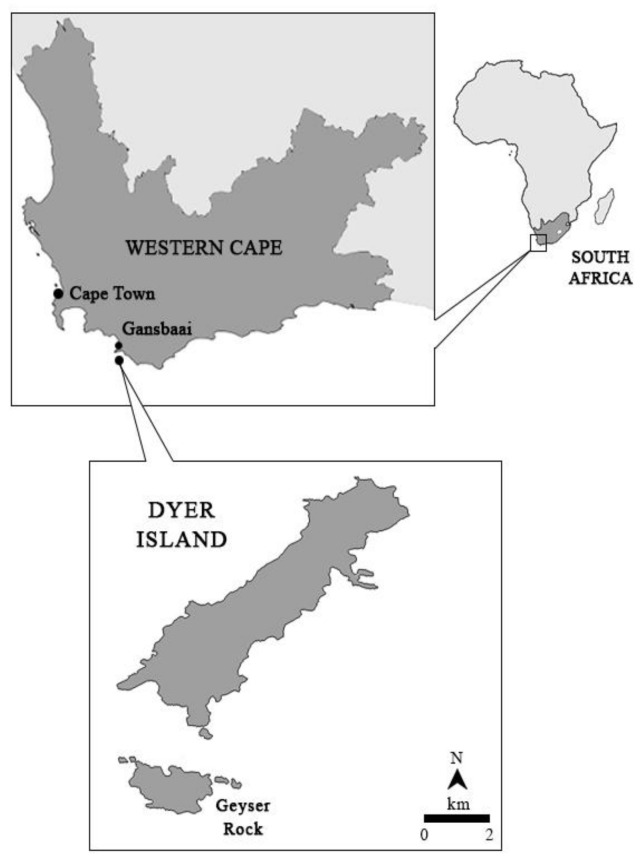
Study area in Dyer Island, South Africa.

Dyer Island Nature Reserve is located on the continental shelf, 7.5 km off Gansbaai, South Africa (34°40′ S; 19°25′ E). The reserve includes two islands: Dyer Island, which is a low-profile island about 1.5 km long and 0.5 km wide, and Geyser Rock, which is about 0.5 km long and 180 m wide (Figure 1). The first is characterized by the presence of African penguins and other seabird colonies, such as cormorants, gannets, and gulls. The second island is home to a colony of Cape fur seals, *Arctocephalus pusillus pusillus* [39]. The 150 m wide shallow channel between Dyer Island and Geyser Rock is known as ‘Shark Alley’. The natural reserve is situated in the Agulhas Bioregion, which is a warm–temperate overlap zone influenced by two main current systems: the cold Benguela Current along the Atlantic coast and the west and warm Agulhas Current along the Indian Ocean [40].

### 2.2. Data Collection

Twelve scientific expeditions were conducted in the study area between 2007 and 2018. Data collection was performed onboard two ecotourism boats in the South African autumn, between March and May. Until 2013, observations occurred aboard a 12 m long boat and, since 2014, aboard a 14 m long boat. Both boats were equipped with a rectangular floating cage made of galvanised steel, housing three observers at a time and moored on the side of the boat. The boats were anchored 50–100 m off Dyer Island at a depth of 14–16 m. Observations comprised more than 40 days at sea and were made ad libitum for 5 to 8 h per day from both the boat and the underwater cage. Sharks were attracted to the area around the boat by chumming [23,41]. The chum was a mixture of seawater, cod liver oil, fish blood, and pilchards. In addition, a tuna head, attached to a buoy and a rope, was used as a floating bait, which was always handled by the same person [14,20]. The Government of South Africa’s regulations for shark cage-diving were strictly followed, and no animal was fed intentionally during the study period.

The sex and maturity stage of the sharks were determined by underwater observations. The total length (TL) of each shark was estimated to the nearest 0.5 m by the comparison of the animal when it approached horizontally, parallel, and close to the 3.5 m long cages. Estimation of TL was always carried out by the same observer. The sex of the sharks was determined by the presence or absence of claspers and confirmed with underwater photographs. Males with a TL > 3.5 m and females with a TL > 4.5 m were considered mature specimens [42]. Identification of individual sharks was based on fin patterns, body scars or mutilations, skin pigmentation, and any useful recognizable markings [43,44].

Environmental conditions, such as sea surface temperature (SST), tides, time of day, sea condition, and cloud cover, were registered in association with the shark sighting. SST was recorded from the boat navigation sensor. The tide’s forecast was used to distinguish the low and high tides. Cloud cover and sea condition were registered using the Okta and Beaufort scales, respectively. The cloud cover was divided into three different categories: clear sky (Oktas 0, 1, and 2), partially covered sky (Oktas 3, 4, and 5), and complete cloud cover (Oktas 6, 7, and 8). The sea condition was determined considering the effects observed at sea, and no data collection was performed under rough weather conditions (Beaufort number equal to or above 4). The time of day was split into different periods: 6.00–10.00 (dawn), 10.00–15.00 (daytime), and 15.00–18.00 (dusk). In addition, each sighting was complemented with data on the lunar phase and El Niño Southern Oscillation (ENSO) phase (El Niño, La Niña, normal) and strength (categorized according to the multivariate ENSO index: weak, moderate, strong, super strong). These were collected from public databases, namely from the Centre for Operational Oceanographic Products and Services [45] and from the Climate Prediction Centre [46], respectively.

### 2.3. Surface Behaviour and Ethograms

The surface behaviour of white sharks was recorded from the boat and from the cage using individual log sheets and, occasionally, a digital photo camera and a digital video camera. The behaviours considered were the ones described by Sperone et al. [20]: bait follow (BF), breach (BR), parading (PAR), visual inspection (VI), tail stand (TSt), spy hop (SpH), tail slap (TSl), repetitive aerial gaping (RAG), and head-up vertical emerging (HVE). The frequency and sequence of these behaviours were registered and then used to create an ethogram that matched the environmental factors at the time. Each ethogram began when the shark approached the bait within a distance of 10 m, and it ended when the shark was more than 10 m away for at least 5 min. The ethograms considered the individual behaviour under the different environmental effects and consisted of one behavioural unit or a sequence of them. In the present study, to assess behavioural complexity, only the data on the number of behavioural units performed by individual sharks were considered.

### 2.4. Data Analysis

The complexity of the ethograms executed was analysed considering the number of behavioural units performed under different environmental conditions, with the assumption that more behavioural units represent more complex ethograms. For the statistical analysis, the number of behaviours was compared according to the sex of the sharks and environmental variables, using the Wilcoxon–Mann–Whitney and Kruskal–Wallis tests for categorical variables. For multiple pairwise comparisons, using the Wilcoxon–Mann–Whitney test, the Bonferroni correction was applied. Numerical variables, such as the length of the sharks, SST, sea condition, cloud cover, moonlight, and ENSO, were analysed through Pearson’s correlation coefficient. All the statistical analyses were performed using software R version 4.0.4 (R Core Team, Austria [47]), and the significance level used was 0.05. In addition, generalised additive models (GAM) were used to model the complexity of ethograms performed by female and male white sharks at Dyer Island in relation to the environmental factors. Two GAM models were developed, one for each sex. Length and maturity stage were also used as predictors. The model was built using the ‘mgcv’ package [47], and the response variable was the number of behavioural units. Since the response variable was a count, a Poison distribution was tested for both models. There was overdispersion (3.03 for females and 2.85 for males), so a negative binomial distribution was used. Predictor variables considered for modelling were numerical: length, SST, ENSO, sea condition, cloud cover, moonlight, and categorical: maturity stage, time of day, and tides.

For numerical predictor variables, the Pearson pairwise correlation was verified to avoid including highly correlated variables (threshold of 0.75), as well as the multicollinearity, through the Variance Inflation Factor (VIF, threshold of 3). None of these variables were highly correlated, so none were excluded from model fitting [48,49]. The number of splines was set to a maximum of 2 for cloud cover and sea condition and 7 for SST and ENSO, in the smooth function, to avoid overfitting.

A backward stepwise selection was used, which consisted of starting with a full model (all predictor variables included, only considering the main effects of the variables), and then removing each predictor variable at each step [50]. To assess whether the removal of the variable decreased model fitting, Akaike’s information criterion (AIC) was used as a goodness-of-fit measure, keeping the model with the lowest AIC, i.e., comparing between models that differed in one explanatory variable (after removing the least significant one). When AIC values differed by less than 2, a chi-square test was applied. If AIC differences were not statistically significant (based on δAIC > 2 or the chi-square test result, with a level of significance of 0.05), the simplest model was kept (following the principle of parsimony, e.g., Burnham and Anderson [51]). The final model was verified (Supplementary Materials Figures S1–S3) with the function “gam.check”, searched for influential data points, checked for observations with Hat values higher than 1.0 [52], and for relationships between residuals and predictor variables (no clear patterns were seen).

## 3. Results

During the 12-year period, a total of 586 ethograms of white shark behaviour, with associated environmental data, were analysed. From these, 58.4% (*n* = 342) corresponded to females’ ethograms, and 41.6% (*n* = 244) of the records belonged to males (Table 1). Additionally, 15.5% (*n* = 91) of the records were from mature animals, while 81.7% (*n* = 479) were from immature ones (out of the total, 2.7%, *n* = 16, of the records did not have any maturity stage associated). When interacting with the bait, 242 ethograms encompassed just one behavioural unit (simple ethograms), while 344 presented a sequence of behaviours (complex ethograms) (Table 1). Hence, a total of 1295 and 731 behavioural units were performed by females and males, respectively, considering complex ethograms.

Primarily, the individual number of behaviours performed towards the bait was compared between males and females, and no significant difference was found (W = 45,453, *p* = 0.054). However, considering the maturity, body length, and the different environmental variables, the response number of behaviours diverged across sexes (Supplementary Materials Figures S4–S13). Overall, mature females performed a higher number of behaviours per ethogram (W = 2503.5, *p* = 0.025) with a positive correlation between the number of observed behavioural units per ethogram and the length of the females, while there were no differences in the behavioural complexity observed in the different maturity stages of the males. However, a negative correlation was found between the number of behaviours and the length of the males, with bigger males presenting fewer complex ethograms. Regarding the environmental variables, females presented more complex ethograms under stronger La Niña events (R = −0.112, *p* = 0.036), cloudy skies (R = 0.209, *p* < 0.001), higher percentage of moonlight (R = 0.120, *p* = 0.027), and during daylight (X^2^ = 8.354, *p* = 0.015). The males only showed a significant response to the moon, behaving with more complexity under higher percentages of light (R = 0.222, *p* < 0.001). Variables such as SST and sea state did not present any significant relationship with the number of behaviours, but males and females responded in opposite ways to these factors. Males performed a higher number of behaviours under higher temperatures and agitated seas, while females behaved with more complexity under lower temperatures and flatter seas.

Since these differences in behavioural responses were noticed between the white sharks’ sexes, the additive relationship of the multiple variables on the individual behaviour of males and females was then analysed through GAM models. The best final models were built with 282 and 205 records for females and males, respectively (Table 2).

Length, SST, ENSO, sea condition, cloud cover, and tides were the explanatory variables that contributed to the final GAM model for female white sharks in South Africa. These 7 variables explained 34% of the deviance of the number of behavioural units per ethogram (R^2^ = 0.103, UBRE = −0.090), evidencing the relationship between these environmental factors and the complexity of the behaviours towards the bait. On the other hand, for males, 8 variables were included in the final model: length, SST, ENSO, sea condition, cloud cover, moonlight, time of day, and tides, explaining 44.7% of the deviance of the male behaviour (R^2^ = 0.377, UBRE = −0.068) (Table 2).

**Figure 2:**
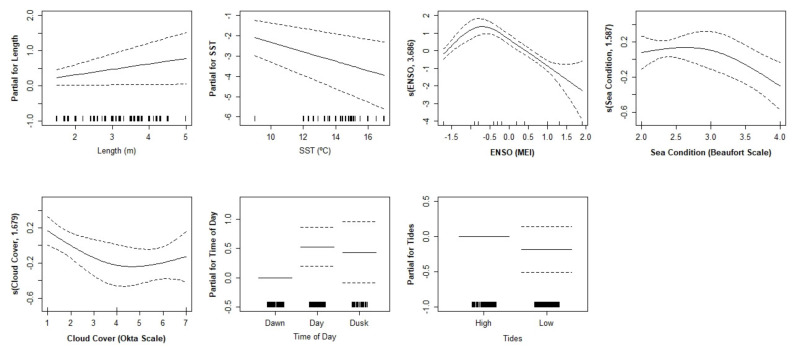
Generalised additive model functions of female white shark behaviour complexity in relation to the explanatory variables. Tick marks above the x-axis indicate the number of observations. The dashed lines represent the 95% confidence intervals of the spline functions. SST = sea surface temperature; ENSO = El Niño Southern Oscillation.

**Figure 3:**
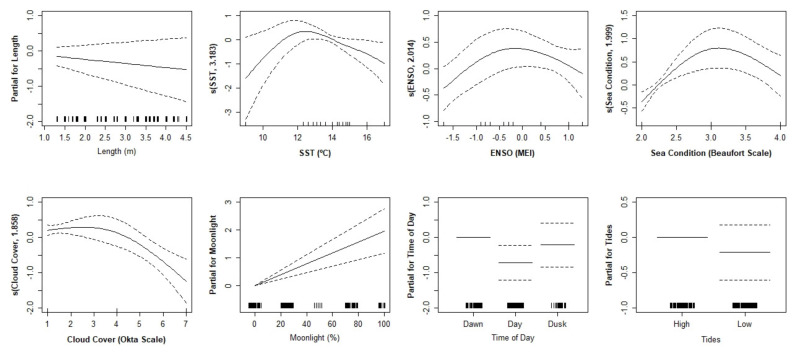
Generalised additive model functions of male white shark behaviour complexity in relation to the explanatory variables. Tick marks above the x-axis indicate the number of observations. The dashed lines represent the 95% confidence intervals of the spline functions. SST = sea surface temperature; ENSO = El Niño Southern Oscillation.

Females and males responded differently to the bait stimulus (Figure 2 and Figure 3, respectively). Larger females and smaller males were likely to perform more complex ethograms. Females also behaved with more complexity under daylight, lower sea surface temperatures, short wavelets, clear skies, under La Niña events, and high tides. Males behaved more complexly at dawn, medium sea surface temperatures, large wavelets, few clouds, high tides, and elevated moonlight.

## 4. Discussion

A combination of univariate and multivariate analysis showed that males and females of *C. carcharias* exhibit differences in their behavioural activity depending on the environmental conditions. Environmental factors rarely occur in isolation and often differ in their level of influence between sex, ontogenetic stage, and geographic location [53]. Here, the influence was analysed factor by factor and using an additive model in order to circumvent this issue. The results of the two statistical approaches should be analysed and interpreted in a complementary manner.

From the 586 ethograms analysed, 59% of them encompassed more than one behavioural unit. It is known that white sharks increase their activity around cage-diving operators, which may impact their energy budget [54]. Overall, when in the presence of bait, more than half of the studied white sharks performed some complex ethograms towards it. It is important to notice that behaviour imposes costs in terms of energy, time, and risks, which must be balanced against survival benefits [55].

Considering the sexual segregation found in white sharks in South Africa [25,26], the number of behaviours performed was compared between males and females. Overall, the complexity of the ethograms was not dependent on sex, showing that data were not biased a priori towards a special group. However, when each environmental factor was analysed individually, considering the sex, all of them showed a sort of influence on the complexity of *C. carcharias* ethograms, confirming the hypothesis assumed.

The Gansbaai *C. carcharias* population has a large proportion of subadults and occasional adults [56,57]. In this study, 82% of the ethograms were executed by immature predators. The total length is the feature used to distinguish shark life history stages and define the maturity stage, which is a categorical variable where different-sized sharks can fit. As such, both variables (maturity stage and length) were used in the analysis. Mature/larger females are statistically likely to be more active around the bait. They probably take advantage of their size, frightening the other sharks from the surroundings in order to increase their opportunity to interact with the bait. As for males, maturity stage and length do not seem to highly influence the complexity of behaviour towards the bait, yet small animals seem to perform a higher number of behaviours per ethogram.

Sea surface temperature is the most studied environmental factor regarding *C. carcharias* behaviour (e.g., [28,58,59]). Females and males seemed to respond differently to variations in temperature. Females clearly decreased the complexity of ethograms with increasing water temperature. Males presented a peak in the number of behaviours around 12.5 °C, with less complexity under temperature extremes, either colder or warmer waters. The white shark is the largest fish with regional endothermy and possibly among the most energy-demanding fishes [60,61]. This predator swimming strategy may maximize net energy gains by reducing swimming and costs with prey attack encounters while increasing the number of behaviours near the bait in order to scavenge and feed on it [61]. In addition, parading (slowly swimming at the surface) was the most performed behaviour by these animals. By performing more complex interactions with the bait under lower temperatures, the shark may avoid spending unnecessary energy. Males and females have different energy requirements depending on their reproductive state. It is also important to highlight that stress can apparently cause lamnid sharks to lose their ability to maintain elevated body temperatures [62]. Tail beat frequency is often used to distinguish stress levels in sharks [63]. This behaviour was not specifically analysed here, but an increment in the general number of behaviours per ethogram is likely to be equivalent in this situation. White sharks are attracted to the bait because they assume a possible debilitated prey can be a source of food. Hence, the complexity of the ethogram may represent the predator’s many attempts to feed.

El Niño Southern Oscillation is the most important coupled ocean–atmosphere phenomenon to cause global climate variability on seasonal to interannual time scales, and it is known to affect the marine ecosystems and marine animals’ distribution and abundance [64]. The temperature variation in the Pacific Ocean impacts the climate across different parts of the world, including South African waters [65,66]. The influences of El Niño and La Niña have been noticed in white shark catches [16,59] and distribution [67,68,69,70]. In the female animals from this study, a strong El Niño caused a reduction in the number of behaviours, while in males, either a moderate El Niño or La Niña event decreased the behaviour complexity. During its cold phase, the La Niña ENSO phenomenon leads to increased easterly wind flow and summer rainfall and reduced coastal sea surface temperatures in South Africa [66]. La Niña is associated with colder waters, and it is possible to verify in the graphs that SST and ENSO follow similar outlines for both of these variables for the different sexes. However, it is important to notice that this phenomenon affects other climate factors. In this way, a possibility is that it is fundamental for the animals to save energy when different environmental factors suffer changes at the same time. Given these phenomena, ENSO also acts as an indirect proxy for wind and rainfall. It would be interesting to analyse the direct impact of these factors on shark behaviour. This was not possible in the present study since data collection was only made under good weather conditions (i.e., that allowed for cage diving). Long et al. [71] and Skubel et al. [58] noticed that El Niño Southern Oscillation might have an impact on prey availability and, consequently, this can indirectly influence white shark behaviour.

The sea state presents a contrary influence on the complexity of female and male white shark ethograms. Females tend to perform a higher number of behaviours when the sea is calm, while males behave more complexly when the sea is slightly rough. The water agitation around the bait gear may mislead the predator into reckoning a live prey or may represent a crypsis advantage for the attack [35]. In Gansbaai, females usually swim near coastal areas, while males spend more time off Dyer Island [25]. This means they may be used to perceiving the agitation in a different way. Male predators may be less used to agitation, so they approach the bait more times, while the females may see the disturbance of the sea as a waste of energy at first. Finding explanations for animal behaviour can easily lead to other topics, such as the ability to learn or individual personality [72,73]. In fact, individual evolutionary fitness is a fundamental aspect influencing animal behaviour.

The cloud cover or the sun glare may condition the detection and observation of white sharks by researchers [30,74]. Still, this factor was analysed, and it was shown to have an influence on white shark behaviour. Considering the additive analysis, females seem to produce a higher number of behaviours per ethogram under clear skies, decreasing them with the appearance of clouds. Males keep their activity high, only diminishing it under a completely covered sky. Pyle and colleagues [30] suggested that less contrasting light conditions cause a darker environment near the surface, which instigates the sharks to swim and surface, with sightings increasing at Farallon Islands. Although this is in accordance with our results from the univariate analysis for cloud cover, the factors do not act in isolation. And so, the additive model of this study presents opposite results, with the cloud cover instigating both males and females to reduce their activity around the bait. Cloud cover is a factor that may be related to the shark’s camouflage ability. Considering that juveniles undertake an ontogenetic dietary shift and most of the ethograms were produced by immature predators, they may not yet fully respond to the benefits of crypticity for predation at the surface [74].

Pyle et al. [30] and Weltz et al. [34] noticed that white shark sightings increase during the new moon, and Fallows et al. [75] observed that both shark attack frequency and seal capture success were significantly higher during the new moon (0–10% lunar illumination). The lack of light and prey movement during the night may make predatory activity difficult; therefore, sharks may tend to execute more behaviours towards the bait during the day in order to guarantee a meal. In the present study, a significant influence of moonlight was found for both males and females, both executing more behaviours when the night had higher percentages of moonlight (full and gibbous moon). Due to the mixed results presented in previous studies, Fallows et al. [75] suggest two primary hypotheses as explanations of the effects of moonlight on predator–prey interactions. The predation risk hypothesis predicts that increases in moonlight will foment predation due to increases in the ability of visual predators to detect and capture prey. In contrast, the visual acuity hypothesis predicts that increases in moonlight will suppress predation due to increases in the ability of visual prey to detect and avoid predators. It is also important to consider that Cape fur seals adjust their behaviour under different lunar conditions to decrease their chances of being hunted [76].

The time of day was strongly connected with light intensity in previous publications; therefore, this factor was split accordingly in this study. Different authors found an influence on the white shark behaviour, mostly associated with the ability of the shark and the prey to detect each other [8,35,77,78,79]. Being a highly opportunistic predator, the white shark willingly scavenges on available carrion, garbage, and fish caught on lines [42]. Considering the bait attraction from the ecotourism platform, scavenging skill is to be expected in this study. Actually, within areas where shark cage diving operations occur, changes in sharks’ diel pattern of habitat use were noticed [21]. Females performed a higher number of behaviours during the day and a lower number during dawn. On the contrary, the males increased the frequency of behaviours during the dawn and decreased during the day. In general, females occur in South Africa year-round, while males are only sighted during autumn and winter [26]. This residency pattern and the bigger female size may contribute to their increased activity in comparison with the males. The latter probably prefer the twilight for a higher interaction, not compromising their detection.

High tides have an indirect influence on the predatory behaviour of white sharks because prey lack haul-out space and move to the water, where they are more vulnerable to attack [18,80]. Here, both females and males performed more complex ethograms under high tides, although the results were not statistically significant. Hammerschlag et al. [35] reported that a shark needs a critical minimum depth in order to approach prey undetected. The water depth from the sampling place was likely sufficient for the animals carrying out a higher number of interactions with the bait.

A relationship between the environmental factors and the white shark behaviour was demonstrated either by univariate and an additive analysis. It is fundamental to consider that the additive effects of several variables may complement elemental analysis, and this should be taken into account in future studies.

## 5. Conclusions

The interaction with cage-diving operators causes an increment in the activity of white sharks by itself, which may impact the energy budget of the animals [54]. This study shows that environmental factors play a major role in the complexity of the activity of white sharks, also. In general, elasmobranchs are able to cope with minor environmental changes in their habitats through compensatory mechanisms that minimize stress [81]. The world’s aquatic habitats are undergoing significant physiochemical shifts due to human-induced climate change [58]. However, many climate change studies ignore animal behaviour, and the understanding of potential effects is critical at this time [58,82]. Cage-diving activities may distract sharks from foraging natural prey [58]. In the context of a changing climate, it is urgent to understand how sharks respond to a fluctuating environment projected under future scenarios. The bait or natural prey consumption may not compensate for the energy expenditure associated with cage-diving interactions [58]. This future source of stress should be studied in order to improve cage diving regulations. Furthermore, this stress can lead some animals to approach fishing vessels. The fishing gear these predators encounter is different according to the fishing season, which is dependent on factors such as, for example, sea conditions, time of the day, and water temperature. Deployment of the fishing gear at specific days or times of the day can reduce or amplify certain behaviours and the risk of the animal getting injured or trapped. In addition, managers could use the environmental factors that influence white shark behaviour to warn fishers when they may have the highest probability of interacting with sharks and encourage them to check their nets more frequently to minimize post-release mortality [83]. The risk of interaction with humans may be slightly higher when local environmental conditions favour the species’ predatory stealth, including the time of the day, sea surface temperature, water depth, and water clarity [84,85]. White shark attacks are also more likely to occur in areas where the ocean is unusually cooler than surrounding areas [86]. In this way, more ecologically responsible approaches are being considered to reduce the risk of shark attacks, which include attempts to understand and exploit patterns in shark behaviour to minimise the likelihood of shark encounters [86,87].

## Figures and Tables

**Table 1:** Description of the ethograms analysed for male and female white sharks. Simple ethograms correspond to those with a single behavioural unit, while complex ethograms include more than one behavioural unit.

	N of Simple Ethograms	N of Complex Ethograms (N of Behavioural Units)	N of Tot Ethograms (N of Tot Behavioural Units)
Females	132	210 (1295)	342 (1427)
Males	110	134 (731)	244 (841)
Total	242	344 (2026)	586 (2268)

N—Number; tot—total.

**Table 2:** Results from the backward selection of GAM models with the explanatory variables and their significance for the model. *p*-values of less than 0.05 were regarded as statistically significant.

Model Parameters	Estimate	Edf	SE	*z*-Value	X^2^	*p*-Value	Deviance Explained (%)	R^2^	UBRE
**Females**									
Intercept	3.632		0.714	5.083		<0.001			
Length	0.156		0.073	2.132		0.033			
SST	−0.232		0.048	−4.814		<0.001			
Time of Day_Day	0.526		0.166	3.170		0.002			
Time of Day_Dusk	0.432		0.260	1.662		0.096			
Tides	−0.183		0.163	−1.124		0.261			
Smoother terms									
ENSO		3.686			66.309	<0.001			
Sea Condition		1.587			4.753	0.049			
Cloud Cover		1.679			4.307	0.067			
**Best model (*n* = 282):** NBehavUnits~length + SST + s(ENSO) + s(SeaCondition) + s(CloudCover) + TimeofDay + Tides							34	0.103	−0.090
**Males**									
Intercept	1.301		0.387	3.366		<0.001			
Length	−0.118		0.101	−1.167		0.243			
Moonlight	0.020		0.004	4.858		<0.001			
Time of Day_Day	−0.719		0.244	−2.941		0.003			
Time of Day_Dusk	−0.212		0.310	−0.685		0.493			
Tides	−0.212		0.197	−1.080		0.280			
Smoother terms									
SST		3.183			9.258	0.039			
ENSO		2.014			7.214	0.035			
Sea Condition		1.999			13.567	0.001			
Cloud Cover		1.858			15.629	<0.001			
**Best model (*n* = 205):** NBehavUnits~length + s(SST) + s(ENSO) + moonlight + s(SeaCondition) + s(CloudCover) + TimeofDay + Tides							44.7	0.377	−0.068

edf—effective degrees of freedom; se—standard error; X^2^—chi-square value; R^2^—R-square value; n—total number of ethograms considered in the model fitting; NBehavUnits—number of behavioural units; SST—sea surface temperature; ENSO—El Niño Southern Oscillation.

## Data Availability

Data for this project are maintained by the University of Calabria and The Sharks Studies Centre—Scientific Institute, Italy. The data are available from the authors.

## Supplementary Materials

The following supporting information can be downloaded at: https://www.mdpi.com/article/10.3390/biology11121735/s1, Figures S1–S3: Generalised additive final model verification; Figures S4–S13: Response number of behaviours by sex, maturity stage, length, sea surface temperature, El Niño Southern Oscillation, sea state, cloud cover, moonlight, time of day, and tides.
