# Supplementary material for: Sex Differences in the Individual Behaviour of Bait-Attracted White Sharks (Carcharodon carcharias, Linnaeus, 1758) Are Linked to Different Environmental Factors in South Africa
Source: Biology (Basel). 2022 Nov 29;11(12):1735. doi: 10.3390/biology11121735 (PMC9774687; doi:10.3390/biology11121735)
Supplement: Supplementary file 1 [file biology-11-01735-s001.zip › biology-2053391-supplementary.pdf]

Supplementary material

a)

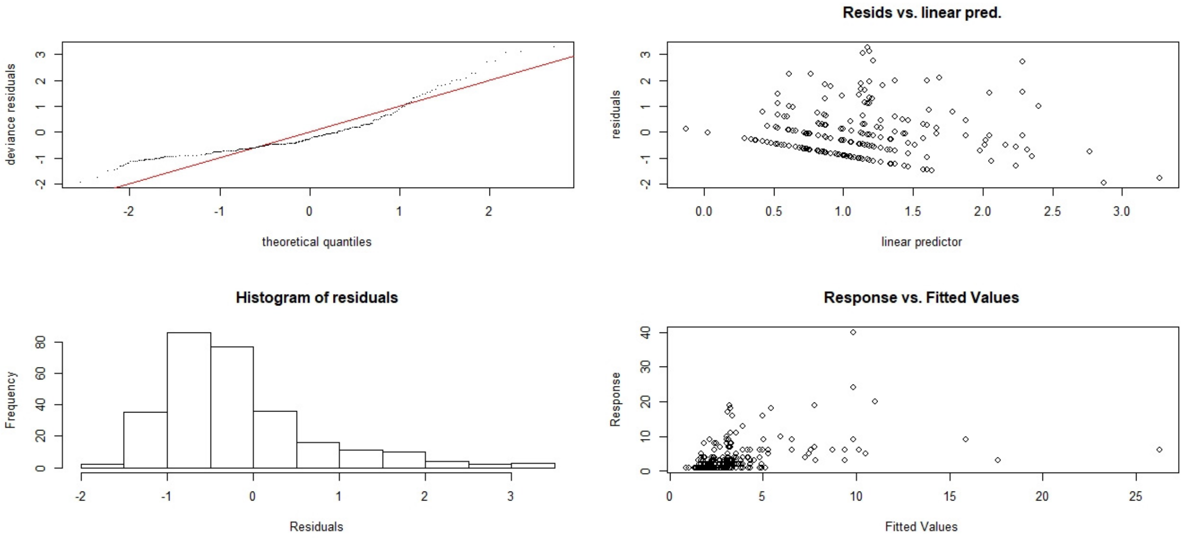

b)

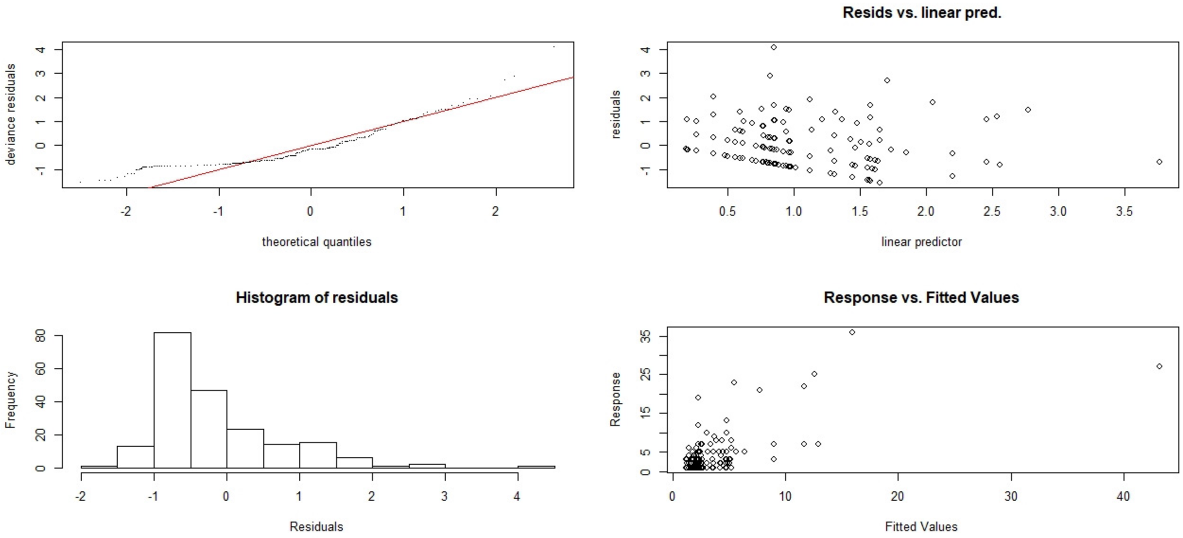

Figure S1. Results from the "gam.check" function of the "mgcv" package, for generalised additive models developed to assess behavioural complexity under different environmental conditions, retrieved from R software. a) Females; b) Males.

a)

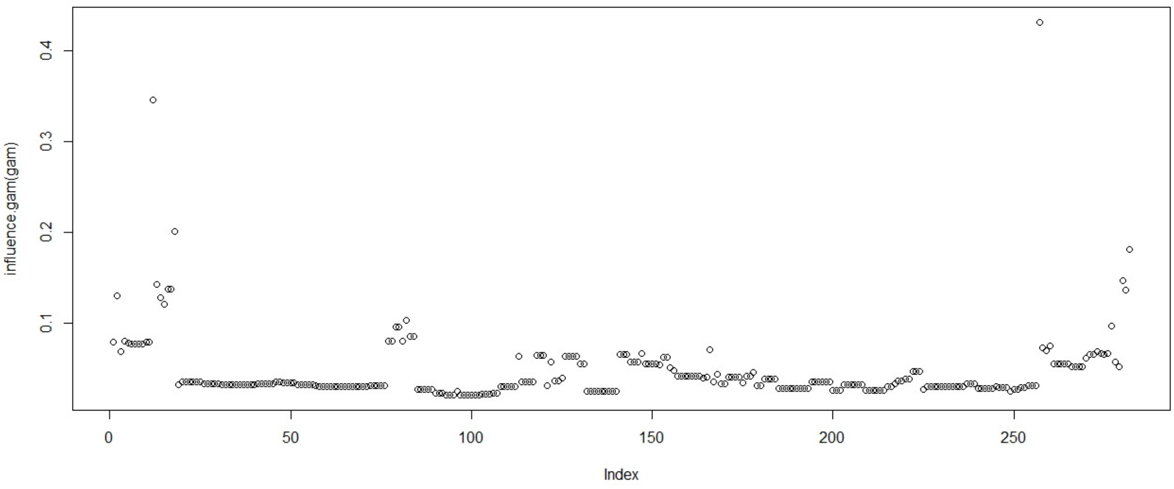

b)

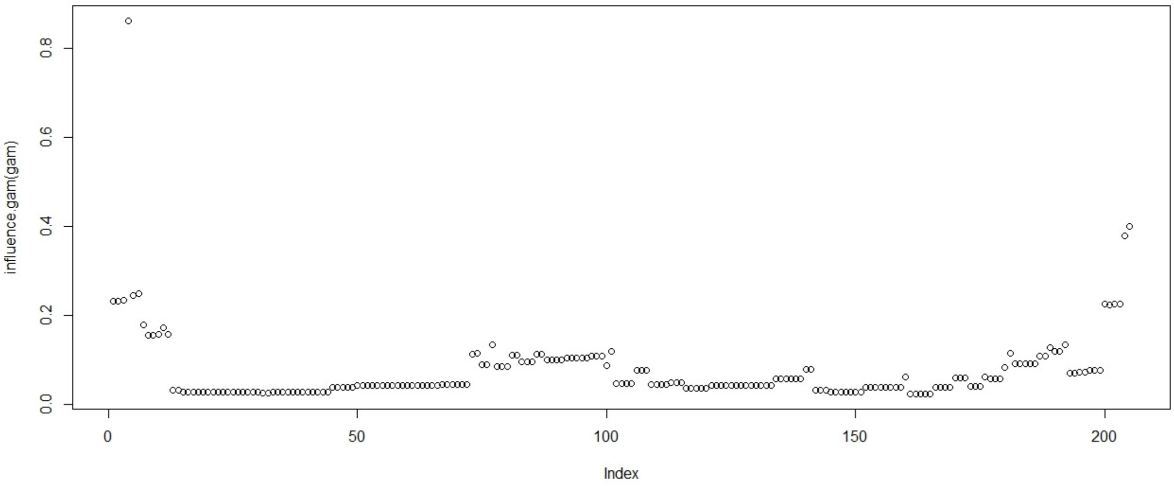

Figure S2. Model verification for influential data points (Hat values), for generalised additive models developed to assess behavioural complexity under different environmental conditions. a) Females; b) Males.

a)

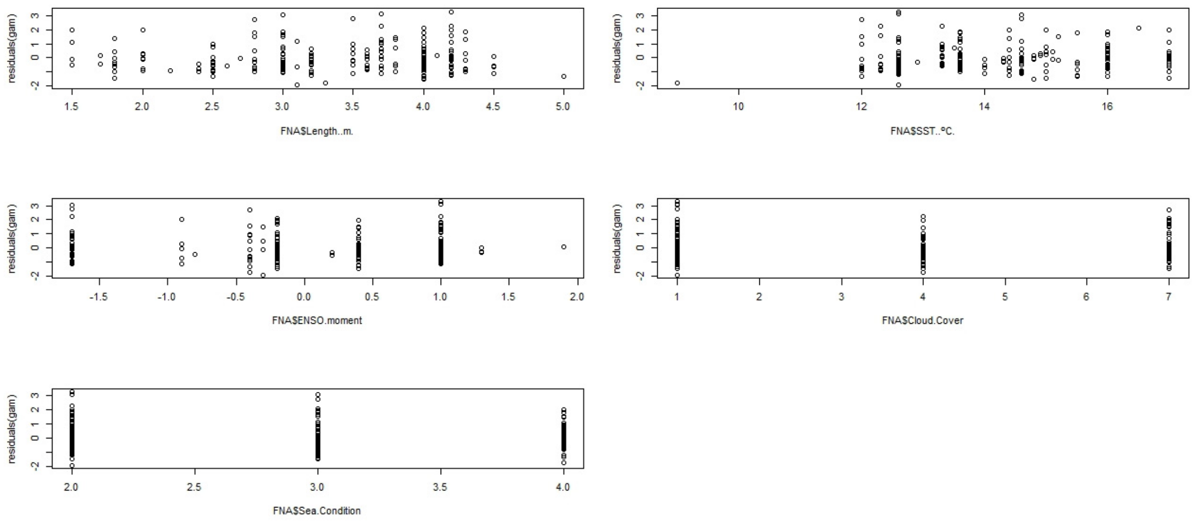

b)

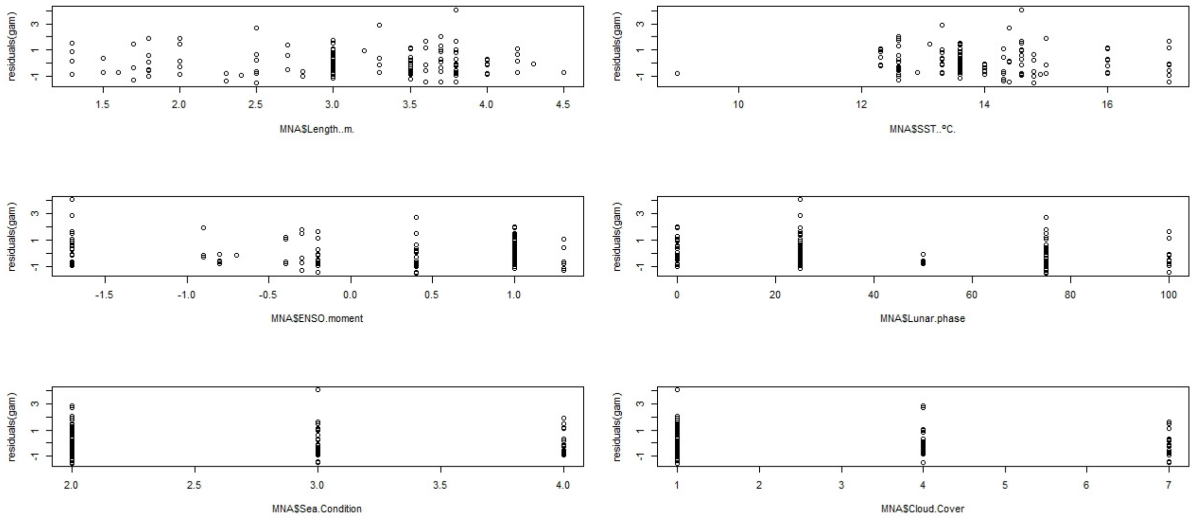

Figure S3. Model verification for relationship between residuals and the predictors, for generalised additive models developed to assess behavioural complexity under different environmental conditions. a) Females; b) Males.

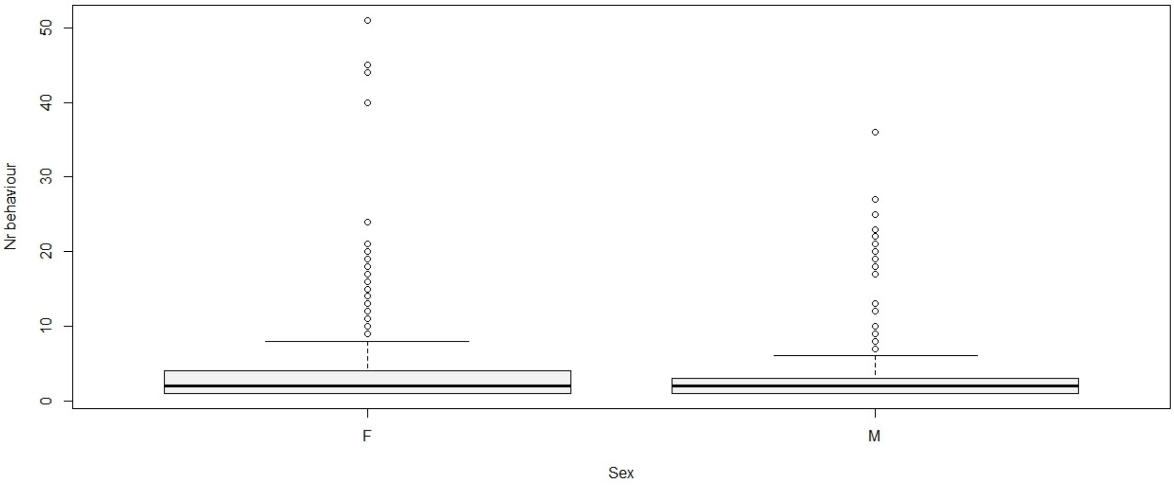

Figure S4. Boxplot representing the number of behaviours executed by females (F) and males (M) white shark.

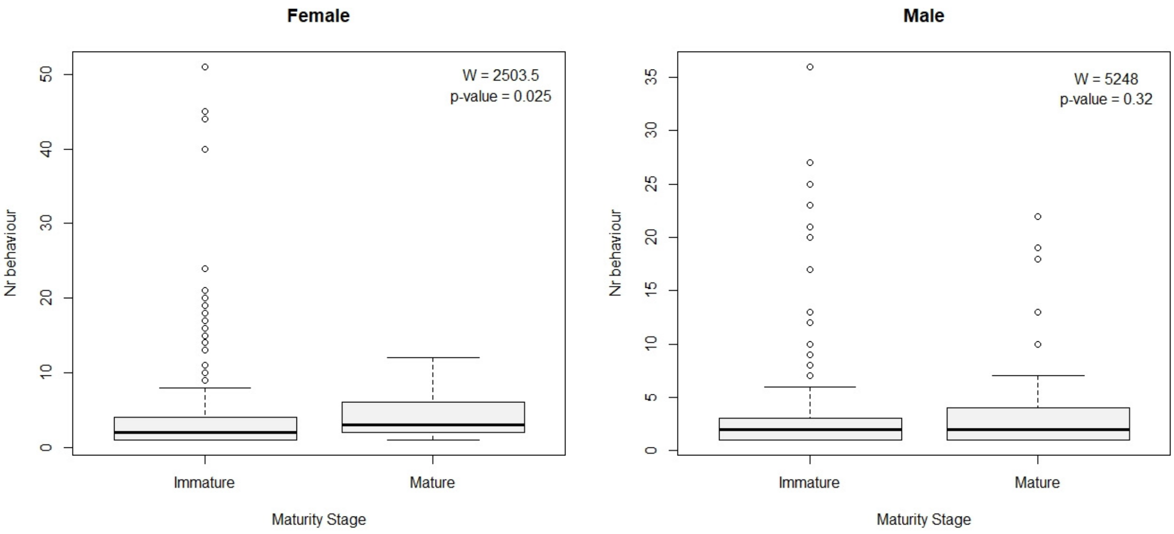

Figure S5. Boxplot representing the number of behaviours performed by females and males' white shark according to their maturity stage. Wilcoxon Mann-Witney Test (W) and *p*-values marked on the top right corners.

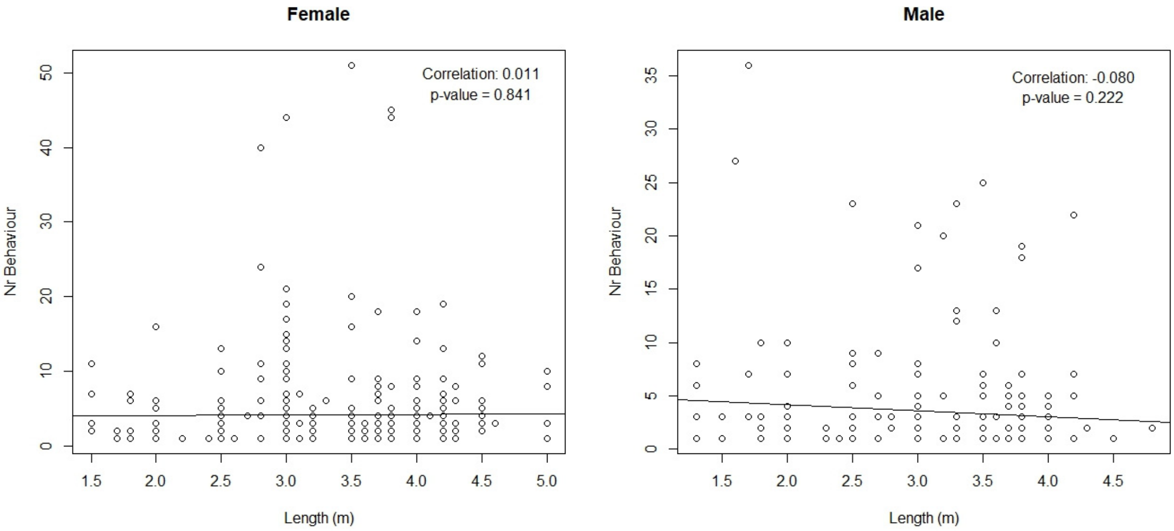

Figure S6. Graph representing the number of behaviours performed by females and males' white shark according to their length. Pearsons' Correlation and *p*-values marked on the top right corners.

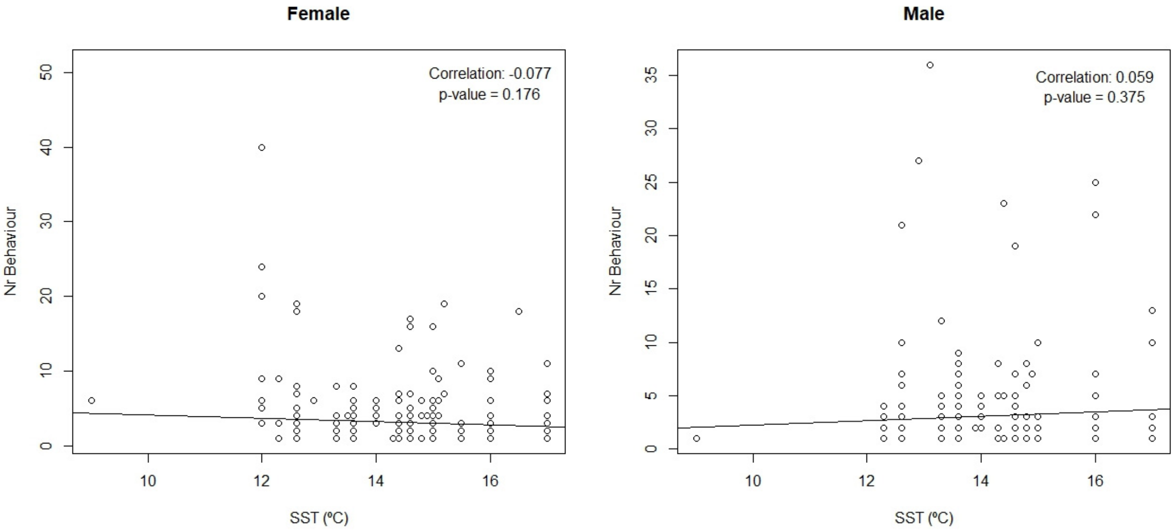

Figure S7. Graph representing the number of behaviours performed by females and males' white shark according to the sea surface temperature (SST). Pearsons' Correlation and *p*-values marked on the top right corners.

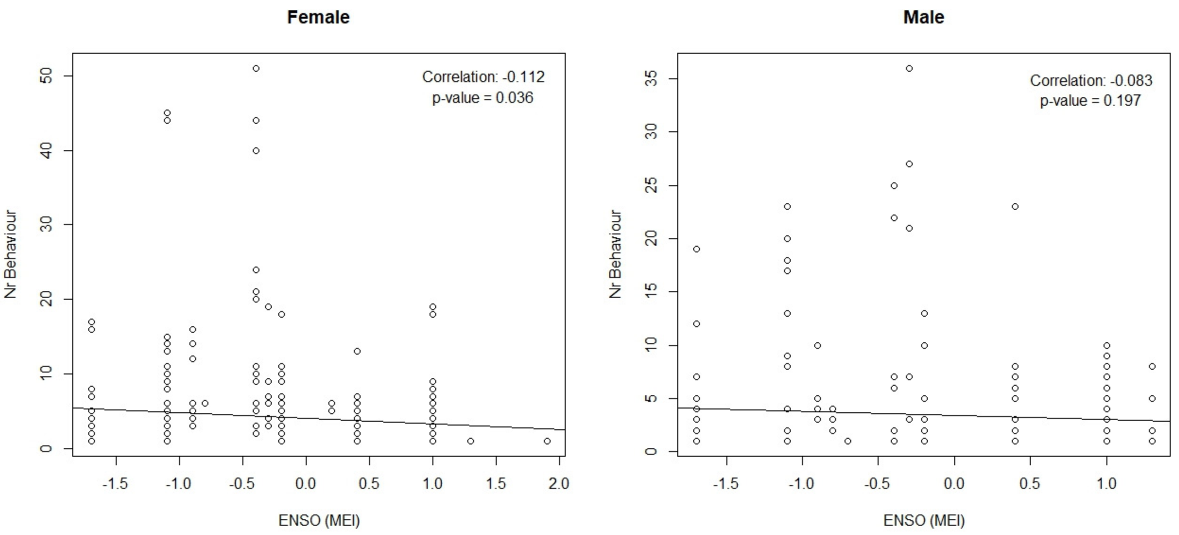

Figure S8. Graph representing the number of behaviours performed by females and males' white shark according to the El Niño southern Oscillation (ENSO). Pearsons' Correlation and *p*-values marked on the top right corners.

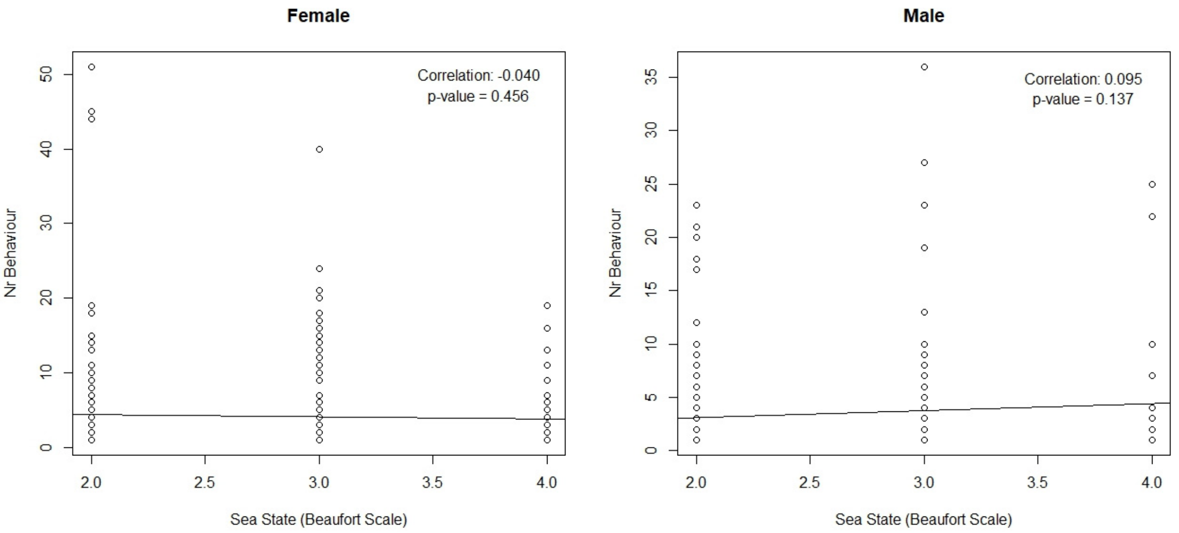

Figure S9. Graph representing the number of behaviours performed by females and males' white shark according to the sea state (2,3 and 4 according to the Beaufort scale). Pearsons' Correlation and *p*-values marked on the top right corners.

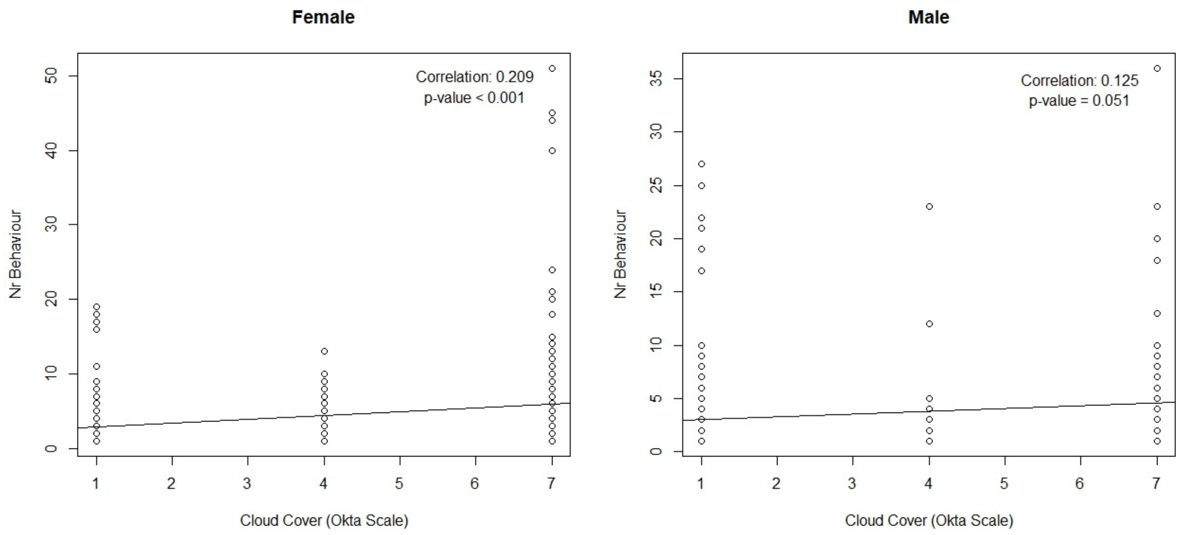

Figure S10. Graph representing the number of behaviours performed by females and males' white shark according to the cloud cover (1 - clear sky; 4 - partially covered sky; 7 - complete cloud cover). Pearsons' Correlation and *p*-values marked on the top right corners.

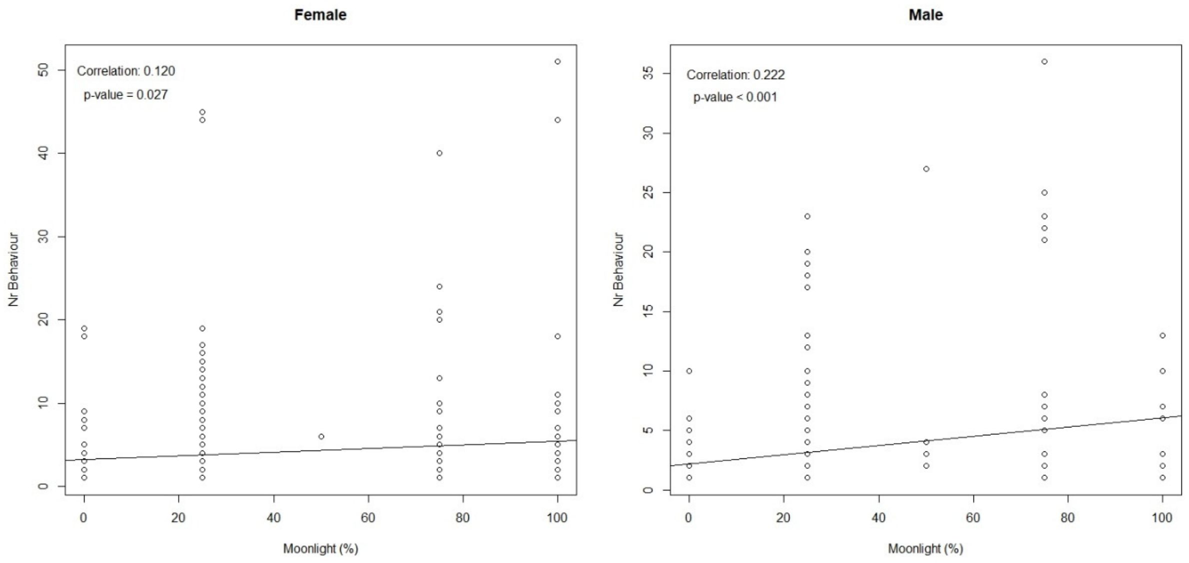

Figure S11. Graph representing the number of behaviours performed by females and males' white shark according to the percentage of moonlight. Pearsons' Correlation and *p*-values marked on the top right corners.

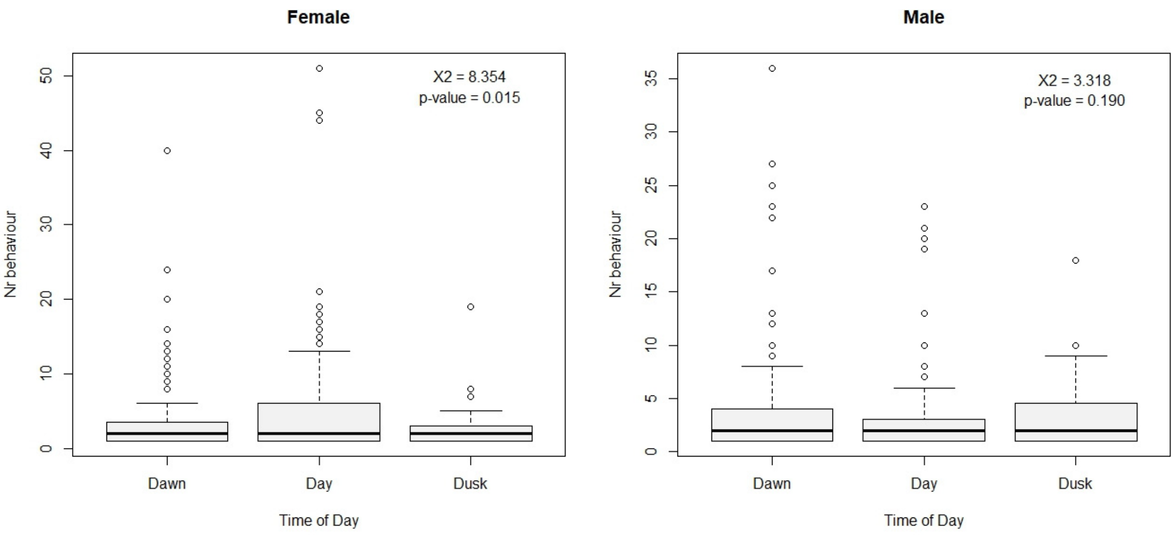

Figure S12. Boxplot representing the number of behaviours performed by females and males' white shark according to the time of day. Kruskal-Wallis Test (X^2) and *p*-values marked on the top right corners.

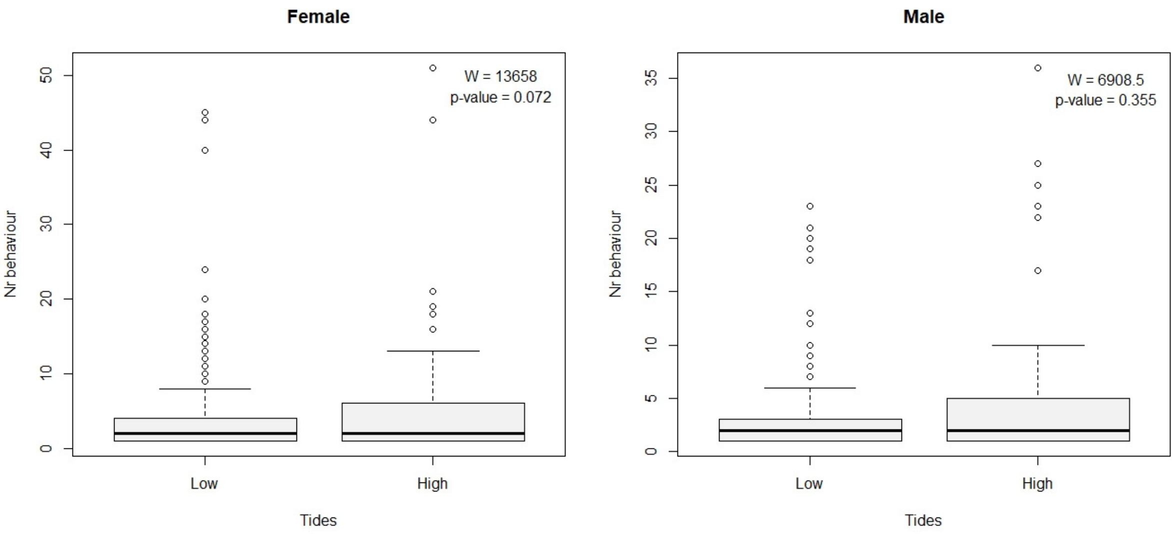

Figure S13. Boxplot representing the number of behaviours performed by females and males' white shark according to the tides. Wilcoxon Mann-Witney Test (W) and *p*-values marked on the top right corners.
